# From Ambition to Action: Navigating Obstacles and Opportunities of “Safe and Sustainable by Design”

**DOI:** 10.1021/acs.est.4c09863

**Published:** 2025-07-18

**Authors:** Joanke van Dijk, Anežka Sharma, Bernd Nowack, Zhanyun Wang, Martin Scheringer

**Affiliations:** † Technology and Society Laboratory, Empa—Swiss Federal Laboratories for Material Science and Technology, Lerchenfeldstrasse 5, 9014 St Gallen, Switzerland; ‡ TEMAS Solutions GmbH, 5212 Hausen, Switzerland; § RECETOX, Masaryk University, 625 00 Brno, Czech Republic; ∥ Institute of Biogeochemistry and Pollutant Dynamics, ETH Zürich, 8092 Zürich, Switzerland

**Keywords:** chemicals management, safe and sustainable by design, SSbD, regrettable substitution, chemical innovation

## Abstract

With the introduction of “Safe and Sustainable by Design” (SSbD), momentum is created in Europe to shift from the reactive (mis)­management of chemicals and materials toward a more proactive design and assessment approach to preventing pollution issues. SSbD is expected to steer the innovation process toward a green and sustainable industrial transition, substitute or minimize the production and use of substances of concern, and minimize the impact on health and the environment throughout the chemical/material life cycle. The European Commission has recommended a framework for operationalizing SSbD, but many open questions remain regarding its feasibility and implementation. Our analysis suggests that despite its potential, the EU SSbD framework in its current form cannot deliver on set ambitions. Suitable assessment methods are not available in many cases, and the complexity and data requirements of SSbD may hinder widespread adoption or result in paralysis by analysis. Moving forward, a more realistic, agile framework, accompanied by clear, simplified methods, and robust support for stakeholders, should be developed to ensure that SSbD principles are fully integrated into practice, leading to truly safer and more sustainable chemicals and materials. We further highlight opportunities to address identified gaps, establish such a framework, and enhance its operationalization.

## Graphical abstract

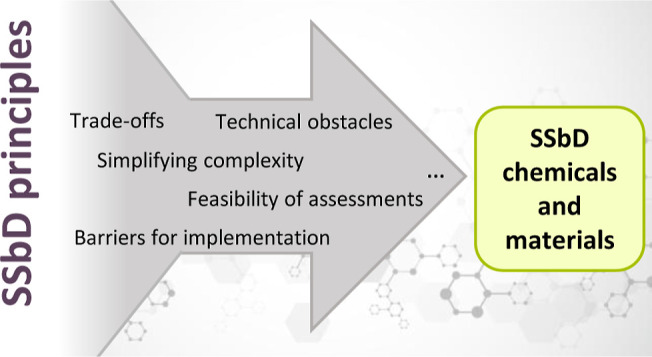

## Introduction

Humanity is facing a triple planetary crisis of climate change, biodiversity loss, and chemical pollution. The current unsustainable production, consumption, and disposal of chemicals and materials contribute to all three parts of the planetary crisis.^1^ The increasing number and quantities of chemicals and materials that have been and are being released into the environment are a major concern for human health^2^ and are driving the global loss of biodiversity.^3^ Moreover, the chemical industry is a major contributor to global greenhouse gas emissions.^4^

Current management of chemicals and materials is unable to deal with these issues.^2^ Foremost, the presumption of “innocence” has often been applied, implying that chemicals and materials are safe unless proven otherwise.^5^ This presumption is problematic, as it has led to the situation that most chemicals and materials were introduced to the market without sufficient safety data but with a high burden of proof for them to be removed from the market. Over the past decades, current management practices have resulted in the widespread use of chemicals and materials for which the hazards and risks are not well-understood.^6,7^ The overwhelming number of chemicals and materials requiring assessment has resulted in a situation in which their hazards and risks cannot be fully assessed and managed in the foreseeable future.

Another major concern with the current regulatory assessment and management frameworks is that they are largely end-of-pipe, based on known problems, and not suitable for promptly dealing with new challenges.^6,8^ For example, end-of-pipe solutions are unable to remove widespread environmental pollution and are expensive to implement.^9^ Furthermore, the phase-out of hazardous chemicals and materials in the past has often resulted in the use of drop-in substitutes with similar chemical structures and, therefore, similar issues, resulting in regrettable substitutions.^10^ Some have highlighted the need to go beyond safety assessments to prevent burden-shifting to other environmental impacts, such as increased carbon and water footprints.^11,12^ Hence, it has been argued that safety and sustainability should be considered at the beginning of the design process of new chemicals and materials, aiming to proactively prevent harm rather than merely reacting to existing pollution. As chemicals and materials are deeply embedded in complex global value chains, their use and management will not only pose a technological problem but also are influenced by the complex interplay between economic investment, societal factors, politics, and technological constraints. Thus, to change the status quo of the production, use, and management of chemicals and materials, a systemic transition with clear incentives is necessary.^13^

Recently, an opportunity for such a step has emerged. In the European Union (EU), the “Chemicals Strategy for Sustainability” (CSS) was published to tackle the triple planetary crisis and enable a green transition.^14^ One particular ambition is to boost the innovation of “Safe-and-Sustainable-by-Design” (SSbD) chemicals and materials. This new concept of SSbD can be seen as a paradigm shift from reactive management toward a more prevention-based approach.^15^ The core of SSbD is that both safety and sustainability aspects should be considered right from the design stage. Similar concepts are also under development elsewhere. For instance, in the US, the Sustainable Chemistry Research and Development Act has been enacted,^16^ among others, to coordinate federal programs and activities in support of sustainable chemistry. In addition, sustainable chemistry is considered by the Organisation for Economic Co-operation and Development (OECD) as an approach to improving chemical, material, and product management by taking a life-cycle approach, and SSbD is considered in the OECD Safe and Sustainable Innovation Approach.^17,18^

To operationalize SSbD, the European Commission has recommended a framework that includes design principles and an assessment procedure covering safety and sustainability dimensions.^19,20^ However, many open questions remain, particularly regarding the feasibility of the EU SSbD framework and its potential for change. Here, we aim to deepen the current discussion of SSbD by raising questions surrounding the SSbD framework. In doing so, we primarily address regulators and scientists; an industry perspective on the SSbD framework would be a valuable contribution to complementing our perspective. We critically analyze and discuss how the “by design” aspect, along with the safety and sustainability assessments of SSbD, constitutes a unique opportunity for the green transition. We evaluate obstacles as well as opportunities of operationalizing the EU SSbD framework and conclude with suggestions for contributions that can be made by scientists, regulators, industry, and civil society groups. We see the many questions arising in connection with the SSbD framework as an opportunity for new thinking and also a constructive discussion among different societal actors about their contributions to the implementation of the SSbD framework.

### An Overview of the European SSbD Framework

The EU SSbD framework (Figure 1) consists of two phases: (1) a (re)­design phase for which guiding principles have been proposed and (2) an SSbD assessment phase. The proposed guiding principles for the molecular, process, and product (re)­design build upon concepts such as green, circular, and sustainable chemistry and address multiple safety and sustainability concerns. The application of the design principles is not mandatory for SSbD. The SSbD assessment consists of four parts: a hazard assessment, a risk assessment for workers during the chemical/material production and processing phase, a risk assessment for human health and the environment during the use phase, and an environmental sustainability assessment. A socio-economic assessment may be conducted as a fifth step. Initially presented as a stepwise approach, it is now recognized that the individual assessments do not always need to be conducted in the same order and can be performed in parallel as information becomes available at various stages of the innovation process of a given chemical or material. It is proposed to communicate the results of the SSbD assessment either by assigning the chemical/material to a class (e.g., poor, good, very good) or by assigning a total SSbD score of all assessment steps.^19,21^

**Figure 1:**
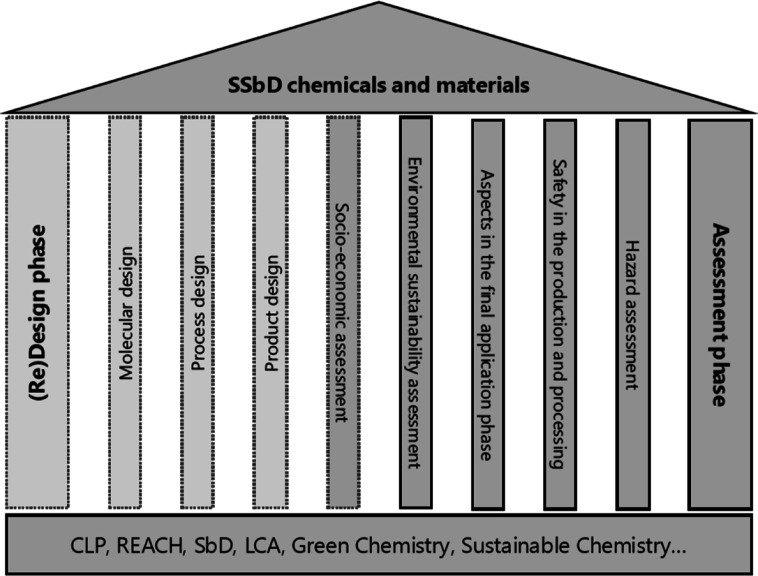
Different components of the EU SSbD framework. The framework builds upon existing concepts and regulations, which can be seen as the foundation of the EU SSbD framework. The pillars illustrate the different elements of the EU SSbD design (light gray) and assessment (darker gray) phases. The application of the design principles is not mandatory. However, for chemicals and materials that are truly SSbD (represented by the roof), the design principles should always be considered; without them, the roof becomes unstable, and the EU SSbD framework would lack significance. Also, the socio-economic assessment is optional, as the primary focus of the EU SSbD framework is on environmental sustainability.

The assessment always starts with a hazard assessment, for which specific cutoff criteria are set to avoid the use of the most harmful chemicals, covering human health (carcinogenicity, mutagenicity, reproductive toxicity (CMR), endocrine disrupting (ED) properties, and specific target organ toxicity after repeated exposure, and respiratory and skin sensitization) and environmental hazards (persistence, mobility, bioaccumulation, ED properties, chronic environmental toxicity, and/or ozone depletion). When a chemical does not exceed the hazard cutoff criteria but has other hazardous properties, the SSbD assessment can continue; however, a lower SSbD score or class may then result from the assessment.

The risks to workers during production and manufacturing, as well as to human health and the environment during the final application phase, are assessed by comparing exposure estimates for these specific life cycle stages against the concentrations considered safe following a standard risk assessment procedure. Environmental sustainability is assessed by conducting a life cycle assessment (LCA).

### Feasibility of the EU SSbD Framework

To evaluate the feasibility of the EU SSbD framework, we critically investigate various technical and methodological aspects and highlight critical issues in Table 1 and discuss them in the following subsections. We also outline several opportunities in the following with the aim of initiating a broader discussion.

**Table 1:** Critical Obstacles with Regard to the Feasibility of the EU SSbD Framework and Its Implementation, as Discussed in This Perspective; for Details, See the Following Sections^a^

topic	element	obstacles	opportunities
feasibility of the EU SSbD framework	design	mainly relevant for new chemicals and materials; does not cover the many chemicals on the market	address the current diversity of chemicals and materials on the market by considering to what extent a chemical or material is needed to deliver a specific function
		lack of green/sustainable production methods	more industry and academic dialogue; create a more supportive environment for the development of methods
		lack of incentives and resources	create a more supportive and enabling environment for the development of methods
		lack of definitions to track progress	define clear and measurable metrics for other end points besides chemical hazard
	safety assessment	questions around defining cutoff criteria for safe/unsafe, limited applicability domain of tools, and combined effects of chemicals	advance AI approaches in combination with more measured chemical property and effects data
		uncertainties and data limitations in projecting future scenarios	develop standardized protocols for developing future scenarios; foster dialogue between different stakeholders to standardize assumptions
	sustainability assessment	challenges in substance testing, uncertainties, and data limitations in projecting future scenarios	advance AI approaches and develop standardized protocols, making assumptions more transparent and consistent
		questions around defining relevant parameters for life-cycle assessments	develop standardized protocols, making assumptions more transparent and consistent
		questions around setting boundaries for life-cycle assessments	develop standardized protocols, making assumptions more transparent and consistent
	integrating safety and sustainability	need for trade-offs	raise awareness, promote transparency, and improve dialogue between different stakeholders
		method issues; weighting subjective and even arbitrary	raise awareness, promote transparency, and improve dialogue between different stakeholders
implementation of the EU SSbD framework	changing the status quo	lack of enforcement of existing regulation	
		continued increase in the use of chemicals/materials	
		SSbD is a voluntary approach	create a supportive environment for SSbD adoption
	assessment relevance	only for specific uses	
	simplifying complexity	challenging to adapt SSbD to industry innovation	raise awareness, promote transparency, and improve dialogue between different stakeholders
		no “one-size-fits-all” strategy	raise awareness, promote transparency, and improve dialogue between different stakeholders

a This compilation is not intended to be exhaustive, and other obstacles and opportunities exist.

## Obstacles to the “By-Design” Aspect

### Mainly Relevant for New Chemicals and Materials

The “by design” aspect of SSbD is an important innovation, acknowledging that the assessment of safety and sustainability needs to come as early in the research and development as possible. However, several challenges need to be addressed in order to make the SSbD framework effective.

The framework does not address the current issue of an already overwhelming diversity of chemicals and materials on the market, but it could potentially add new ones, increasing the complexity and diversity of the assessment task. It would be crucial that the design phase also consider to what extent a chemical or material is actually needed in order to deliver a specific function, for example, by applying the essential-use concept.^22–24^ In other words, uses that are not needed from a technical/functional point of view—i.e., unnecessary and unsustainable uses—should be avoided.^25,26^ Also, it would be desirable for several companies to jointly develop SSbD chemicals and materials that can replace several hazardous chemicals across the same applications.

### Lack of Definitions to Track Progress

The design of better chemicals has been promoted before through concepts such as Green Chemistry, Sustainable Chemistry, and Safe-by-Design. However, the widespread adoption of these concepts has been limited by several obstacles that are also relevant for SSbD. The SSbD framework does provide hazard cutoff criteria that address problems in existing concepts arising from a lack of clear definitions of “safe”.^27^ However, such criteria do not exist for sustainability, as “sustainable” is a very broad concept, and there is a need for clear and measurable metrics for other end points besides chemical hazard. This lack of agreed-upon metrics makes it difficult to incorporate the sustainability dimension into the design process and to track progress. Another important limitation is that, so far, mostly criteria for improved chemical processes have been adopted, but not criteria for improved chemicals.^28^

### Lack of Incentives and Resources

Clear principles and incentives are key to driving sustainable innovation in industry,^29^ while purely economic interests tend to result in the continuation of existing practices, even those that are polluting.^30^ Current manufacturing systems are extensively interconnected and operate at a global level, making changes slow and difficult. Moreover, a lack of resources can hamper the implementation of new chemical design processes, disproportionately affecting small businesses.^31^ Meanwhile, SSbD will, as it currently stands, remain a voluntary framework. This emphasizes the importance of developing proper incentive systems covering entire supply chains that clearly favor the adoption of SSbD and prevent SSbD from being perceived solely as a superficial label, not only within the EU but also globally.

## Safety Assessment

### Defining Cutoff Criteria, Limited Applicability Domain of Tools, and Combined Effects of Chemicals

Currently, in the EU SSbD framework, cutoff criteria are only set for the hazard assessment. These cutoff criteria are largely grounded in existing legislation, such as REACH and CLP, which is an advantage of the framework. However, it would be critical to establish a mechanism within the SSbD framework to regularly evaluate and update the hazard cutoff criteria as the science advances.

Meanwhile, several other technical matters may hamper the operationalization of the SSbD framework on the hazard side. First, while many in-silico tools exist for estimating chemical properties, they are not widely applicable to all hazard end points for all types of chemicals and materials.^32,33^ To overcome this technical obstacle toward implementing SSbD as early in the innovation process as possible, considerable efforts are needed to greatly expand the scope and applicability domain of in-silico tools in a strategic manner, e.g., by developing high-throughput testing and carefully selecting target chemicals for testing in order to efficiently generate large data sets for refining existing tools and developing new ones.^33,34^ Also, approaches using artificial intelligence (AI) provide promising starting points and perspectives but need to be grounded in sufficient measured data.^35,36^

Also, the exposure and risk assessment aspects of the SSbD framework partly rely upon different types of existing legislation, including REACH (Regulation (EC) No 1107/2006) and Occupational Safety and Health (OSH) (Framework Directive 89/391/EEC).^37^ Thus, in principle, the integration of exposure and risk in the SSbD framework would not pose a large new burden to manufacturers. However, the current exposure and risk assessment methodologies need to be improved before they can deliver on what is expected under SSbD, as they have fundamental limitations—such as limited applicability domains and lack of consideration for mixture effects—which have been discussed previously.^38,39^ Also here, AI approaches could help; however, to exploit the power of such approaches, more high-quality data are needed to feed into the models.

Furthermore, some types of substances are difficult to test, e.g., chemicals with low water solubility, and established regulatory testing is unsuitable for identifying certain hazardous properties. Therefore, new experimental protocols should be developed to comprehensively identify relevant hazards for the wide variety of chemicals and materials on the market or soon to be marketed.^40^ It should also be generally recognized that risk assessments are ineffective for highly persistent or bioaccumulative chemicals for which exposure can only be slowly reversed, even if exposure sources were eliminated immediately. In other words, when risk assessments indicate a risk for highly persistent chemicals and materials, it may already be too late to take action.^5^ Furthermore, it should be noted that the safe use of individual chemicals does not guarantee the safe use of chemicals in general, due to their accumulation and combined effects.^41–43^ This is another aspect not currently covered by the EU SSbD framework.

### Uncertainties and Data Limitations in Projecting Future Scenarios

As a general point, uncertainties exist at all stages of the innovation process (and can be handled to some extent by sensitivity, uncertainty, and scenario analyses). Progress in the development of a chemical or material will decrease some of these uncertainties.^44^ However, there are some types of uncertainties that need to be given particular attention in the SSbD framework with its strong focus on early innovation stages. Great uncertainties exist in projecting future scenarios. The estimation of emissions has been identified as the least developed step of risk assessment, as the necessary information is often not available for current uses, let alone for future scenarios.^45,46^ More specifically, in the context of SSbD assessments, it is unclear how accurate the exposure estimates will be in ensuring “safe uses”. The required information about a product’s identity, type, use, and composition is limited or missing at the early design phase, as many new uses have not yet been identified. Moreover, data related to the end-of-life stages are difficult to obtain at any given point during the innovation process due to scientific and practical limitations.^47^

## Sustainability Assessment

### Challenges in Substance Testing, Uncertainties, and Data Limitations in Projecting Future Scenarios

In the EU SSbD framework, sustainability should be ensured by minimizing the environmental footprint of chemicals and materials—particularly with regard to climate change, resource use, and the degradation of ecosystems and biodiversity—by adopting a life-cycle perspective.^19^ Several sustainability aspects are already covered under existing legislation, such as the EU Ecolabel regulation (EC No 66/2010), which presents a voluntary environmental labeling scheme that requires scientific data on the whole life cycle of a product. Thus, implementing the SSbD framework could help manufacturers meet the requirements under the EU Ecolabel regulation or vice versa, with the EU Ecolabel regulation providing additional incentives for implementing the framework.

Currently, the SSbD framework recommends LCA for sustainability assessments. After decades of development, LCA has matured, with existing ISO guidelines standardizing the major steps of an LCA: (i) goal and scope definition, (ii) life-cycle inventory, (iii) life-cycle impact assessment, and (iv) result interpretation.^48^ More specifically, the European Commission has recommended the use of the Product Environmental Footprint (PEF) and Organization Environmental Footprint (OEF) as the full LCA methods for SSbD assessments.^19^

There are, however, many known challenges to applying LCA methods. All LCAs include inherent uncertainty due to the variety of data sources, assumptions, and gaps, and the data interpretation and decisions that are made after conducting an LCA remain dependent on personal values and opinions.^49^ Concerted efforts are still needed to fill the large data gaps with regard to the life-cycle inventory of chemicals and materials, including completing existing ones (e.g., by addressing production emissions).^50^ Novel technologies such as deep learning may provide some new opportunities to do so.^51^ However, while more use of LCAs will result in more data being available and more cases being available for exploitation by machine-learning approaches, fundamental principles for conducting LCAs in the context of SSbD, in particular regarding the problem of subjectivity in many basic assumptions, still have to be established. Clear guidance needs to be developed and widely implemented for SSbD to streamline the process of assessing and communicating LCA-inherent uncertainties in a consistent, transparent, and accessible manner.

Another gap in the SSbD framework concerns chemicals as an important factor in the triple planetary crisis of pollution, climate change, and biodiversity loss.^52^ SSbD does not specifically address biodiversity conservation. Novel chemicals have been identified as an issue of concern to biodiversity protection,^53,54^ and SSbD does not appear to address this as it only indirectly covers biodiversity by considering aspects such as climate change and land use. That said, ongoing and future efforts to study, assess, and address the biodiversity impacts of chemicals are both necessary and warranted.

### Defining Relevant Impacts of LCAs

It should be avoided that impacts are assessed more than once under SSbD. For example, risks to human health with regard to cancer development are currently considered under both steps 2 and 3 (occupational risk assessment and human health risk assessment) as well as in the environmental sustainability assessment. It may be more effective to cover such human health risks in the risk assessment steps only in order to prevent unnecessary complexity and duplication of work in data interpretation. Overall, to ensure the quality of LCAs, broad and open scientific discussions about the methodology and transparency of data are required,^49^ highlighting the need for the further development of guidelines and rules for their use in the context of SSbD.

### Setting System Boundaries for LCAs

LCAs can be performed with different system boundaries, as is also explained in the SSbD Methodological Guidance of the EU Joint Research Centre.^21^ Generally, the choice of the system boundaries is rather subjective, leading to varying outcomes that could potentially support misleading conclusions.^55,56^ Furthermore, there has been an ongoing discussion about shifting from relative improvements (safer and more sustainable) to absolute enhancements (safe and sustainable), with the aim of ensuring that chemicals and materials are produced and used globally without exceeding planetary limits, as aspired to by Rockström et al.^57^ However, absolute sustainability boundaries would still have to be defined in order to establish these aspired SSbD cutoff criteria, which is a highly complex task due to many open questions in allocating planetary limits across regions, sectors, and generations. This complexity may present a barrier to the sustainable design of SSbD chemicals and materials. While certain impacts (climate change, ocean acidification, damage to the ozone layer, etc.) are globally defined, others vary depending on regional scales. This variability complicates the allocation of boundaries and adds complexity to establishing absolute limits.^58^ The SSbD framework may start with the more conventional comparative analysis first, while continued research into methods for absolute sustainability assessment is conducted.

## Integrating Safety and Sustainability

The integration of safety and sustainability aspects in the SSbD framework can be contentious, raising the issue of addressing trade-offs between safety and sustainability. It has been highlighted^11^ that substituting hazardous chemicals with safer alternatives could potentially result in burden-shifting due to the increased carbon and water footprints of these alternatives. Different approaches to combining safety and sustainability have been presented,^59^ highlighting multiple challenges and emphasizing that this process will need to be continuously adapted as new methods and data become available.

Importantly, an approach is needed that involves different stakeholders, including industry, academia, civil-society groups, and governments, to prevent harmful trade-offs.^60^ Shifting from fossil- to biobased materials is not generally desirable, for example, because there are trade-offs between lower greenhouse gas emissions and other environmental impacts, such as biodiversity loss and water stress from agricultural practices.^61,62^ Additionally, the safety aspects of these alternative technologies are also not necessarily well-understood.^63^ Ideally, relevant societal actors should come together to discuss these gaps and trade-offs and decide which negative effects are acceptable and why. As this will not be realistic, future research should focus on developing a pragmatic approach—based on methods such as Multiple-Criteria Decision Analysis^64^—to facilitate such a multistakeholder process without causing paralysis by analysis.

## Implementation of SSbD

### Changing the Status Quo

Several policy targets have been set at both the regional and global levels to better manage chemicals and waste and minimize their effects on human health and the environment. However, these targets have not been achieved to date.^2^ With the SSbD framework, there seems to be a general assumption that the current crisis of chemical pollution can be resolved if more and better data were available. However, many challenges also lie within the production and consumption systems in place, which are not addressed by the SSbD framework in its current form or by any regulation at all. While the SSbD framework appears to touch upon many urgent issues, as discussed above, it remains to be seen how effective it will be, as a voluntary premarket approach, in driving the much-needed change toward proactive chemical and material management. In particular, over the past decade, there has been an increase in the volumes of known hazardous substances used in the EU,^65^ and the continued marketing of hazardous substances as a result of a lack of enforcement has been highlighted.^66^ Under the SSbD framework, hazardous substances will not be given the label of “SSbD” but can still be marketed. This is a gap that requires further improvements in chemical regulation and management, including enforcement.

### Assessment Relevance

When a chemical is designed and registered for a specific use under regulatory frameworks, it is possible that it may be used in alternative applications over time, impacting the emission routes, human and environmental exposure, and other variables. The SSbD case studies published so far are restricted to a very specific use case (e.g., a specific plasticizer in a sealing gasket made of a plastic liner with elastomeric properties placed below the metal cap in glass jars^67^). The result of the SSbD assessment is then valid only for this particular application, and no general classification about the safety and sustainability of the targeted chemical is possible. The assessment would need to be repeated if a significant new use of the chemical or material is introduced, also considering the combined exposure routes from the different uses of the same chemical, which requires significant resources and could easily lead to paralysis by analysis. It remains to be seen whether a more general SSbD assessment of a chemical is possible or not, going beyond very specific case studies.

### Simplifying Complexity

Under the SSbD framework, the industry will mainly be stimulated to make use of already existing concepts and tools. However, this requires additional data and a high level of (very) specialized expertise, which might result in inaction, as SSbD could lead to onerous additional work. If every SSbD assessment requires resources similar to the SSbD case studies published,^67^ industry might resist its implementation as the resources to perform such assessments are not available, particularly for small- and medium-sized companies. The perspectives of different industrial value chains should be considered in this context.^35^

Thus, simplification of the SSbD assessment is critical for its long-term and large-scale operationalization. This could be achieved by including simpler metrics to measure a chemical’s sustainability footprint when more advanced and detailed data are not available.^51^ Moreover, companies often have different priorities at various stages of innovation (i.e., the stage-gate model), and therefore, it would not be logical to apply the same SSbD assessment at each stage of innovation. For example, businesses may first decide on the molecular structure, which has major implications for hazards, and then move on to designing the manufacturing process, which has major implications for sustainability but not for the hazards of a given chemical or material. Thus, an efficient SSbD assessment procedure would be built upon the focus of each innovation stage. However, there is no one-size-fits-all simplification strategy, calling for clear guidance on conducting and communicating simplified assessment approaches.^68^

The industry has been shown to be capable of finding innovative solutions to pressures of all sorts from competitors, customers, and regulators. Properly designed environmental standards can have economic benefits for industry, as they trigger innovations that lower the total cost of a product or improve its value.^69^ Ultimately, the effectiveness of SSbD depends on addressing its inherent challenges and fostering both regulatory support and industry commitment. It will thus be crucial for further developments in SSbD to address the issues we have outlined here and strive toward simplified yet effective methods, but also collections of tools combined with decision-support frameworks, such as those currently developed within projects such as PARC^70^ or SUNSHINE.^71^

### Way Forward

Momentum is emerging for SSbD to shift the current mismanagement of chemicals and materials toward a proactive design and assessment approach. While promising overall, the EU SSbD framework remains abstract in its current form, with specific parts lacking clear guidance for users. It is essential to ensure that the outputs generated by the SSbD framework are meaningful and not used as superficial labels but truly demonstrate that the developed chemical and material is better for society and the environment.

Different stakeholders have distinct roles in advancing SSbD. For example, scientists can generate data, simplify methods, and point out inherent limitations and uncertainties, particularly regarding trade-offs and choices to be made, as there will necessarily be conflicting objectives. Another critical task for scientists will be the development of methods and tools with broader applicability domains. Scientists should also work to tailor the SSbD framework to the different steps of the chemical product development process, which will require collaboration with other stakeholders, especially with industry.

Regulators should develop incentives that create a level playing field, ensuring SSbD is not only adopted by large companies as a result of resource constraints faced by smaller entities. These incentives should guarantee long-term commitment (>5 years) to the EU SSbD framework. Moreover, regulators should also provide adequate funding and support for scientific work, including topics that are often overlooked due to limited publishing opportunities but that are highly important, such as the generation of experimental data with standardized tests as a basis for in-silico property and hazard assessment methods.

Industry should actively collaborate with research institutions on case studies, publish their data, and share information about the product development process.

Civil society organizations can add relevant dimensions by providing independent assessments of SSbD and by offering viewpoints from consumers, for example, regarding the assessments’ transparency and clarity, and by proposing case studies.

Moreover, in order to avoid personal biases affecting the priorities in SSbD assessments, all parties involved should collaborate to create clear guidelines, discuss trade-offs and system boundaries, and decide which types of impact should be prioritized over others and why.

## Abbreviations

CLP: classification, labeling, and packaging
CMR: carcinogenic, mutagenic, toxic for reproduction
CSS: Chemicals Strategy for Sustainability
ED: endocrine disrupting
EU: European Union
LCA: life cycle assessment
OECD: Organisation for Economic Co-operation and Development
OEF: Organization Environmental Footprint
PEF: Product Environmental Footprint
REACH: registration, evaluation, authorisation and restriction of chemicals
SSbD: Safe and Sustainable by Design
